# A study on the impact and buffer path of the internet use gap on population health: Latent category analysis and mediating effect analysis

**DOI:** 10.3389/fpubh.2022.958834

**Published:** 2022-11-02

**Authors:** Yuanyuan He, Lulin Zhou, Xinglong Xu, JunShan Li, Jiaxing Li

**Affiliations:** ^1^Department of Management, Jiangsu University, Zhenjiang, China; ^2^Department of Economics and Management, Jiangxi University of Chinese Medicine, Nanchang, China; ^3^Metropolitan College, Boston University, Boston, MA, United States

**Keywords:** internet use gap, internet information dependence, population health, latent category analysis, regression analysis

## Abstract

**Background:**

The development of Internet information technology will generate an Internet use gap, which will have certain adverse effects on health, but internet information dependence can alleviate these negative effects.

**Objective:**

This article is to demonstrate the negative impact of the internet use gap on population health in developing countries and to propose improvement paths.

**Methods:**

This article used the 2018 China Family Tracking Survey database (*N* = 11086). The research first used Latent class analysis (LCA) to identify potential categories of users with different Internet usage situations, then used the Bolck, Croon, and Hagenaars (BCH) method to perform latent class modeling with a continuous distal outcome, and finally built an intermediary model about Internet information dependence based on the model constraint function in Mplus software.

**Results:**

(1) The Internet users can be divided into light-life users (C1: *N* = 1,061, 9.57%), all-around users (*N* = 1,980, 17.86%(C2: *N* = 1,980, 17.86%), functional users (C3: *N* = 1,239, 11.18%), and pure-life users (C4: *N* = 6,806, 61.39%). (2) We examined individual characteristics, social characteristics and different living habits, and health differences between the latent classes. For example, there are certain structural differences on the effect of different categories of Internet use on health (C1: *M* = 3.089, SE = 0.040; C2: *M* = 3.151, SE = 0.037; C3: *M* = 3.070, SE = 0.035; C4: *M* = 2.948, *SE* = 0.016; *P* < 0.001). (3) The Internet use gap can affect health through the indirect path of Internet information dependence, and some of the mediation effects are significant. When the functional user group (C3) was taken as the reference group, the mediating effect values of light-life users (C1) and all-around users (C4) on health were −0.050 (SE = 0.18, Est./SE = −3.264, *P* = 0.001) and −0.080 (SE = 0.010, Est./SE = −8.412, *P* = 0.000) through Internet information dependence, respectively. However, the effect of categories on health was not significant after adding indirect paths.

**Conclusion:**

The Internet use gap has a significant effect on health, and Internet information dependence plays an intermediary role in this effect path. The study proposes that attention should be paid to the diversified development of Internet use, the positive guiding function of Internet information channels should be made good use of, and the countermeasures and suggestions of marginalized groups in the digital age should also be paid attention to and protected.

## Introduction

With the innovation and transformation of information technology and the in-depth expansion of application space, the Internet not only affects social progress and economic development but also changes the production and lifestyle of the population. According to the “Digital 2021: Global Overview Report” released by the DataReportal research institute, the current global Internet users account for 59.51% (4.66 billion/7.83 billion) of the global population, 5.22 billion mobile phone users, and the penetration rate is 66.6%. Internet users spend nearly 7 h online every day, and the penetration rate and application rate of the Internet have reached a new high (1). As the largest developing country, China has 1.032 billion Internet users, and its Internet penetration rate is as high as 73.0%. The daily Internet online time exceeds 4 h, which is much higher than the overall level of the global Internet penetration rate (The 49th “Statistical Report on Internet Development in China,” 2022), and the status of China's Internet as the world's largest network has become increasingly prominent (2). Thanks to the accumulation and iteration of basic Internet resources and the rich innovation of digital application technology services, the Internet has penetrated people's daily life from multiple dimensions such as study, work, social interaction, and entertainment. The frequency of activities and information dependence have been continuously strengthened. The digitalization of the earth's inhabitants is irreversible, and the Internet is one of the social determinants of health (3). Improving population health is one of the important goals of digital governance(4).

The results of Internet development have created differentiation, gaps, and even gaps between the digital and non-digital groups, and this differentiation is called the Digital Divide (5). The concept of the Digital Divide proposed by the OECD includes two dimensions, namely, “Whether Internet access (Have)” and “Whether there is a difference in Internet use (Use)” (6, 7). The “Internet use gap” (IUG) is the difference in Internet use and one of the important manifestations of digital inequality (8). The current Digital Divide is mainly manifested in the differences in digital skills and user behavior. According to the different purposes of using Internet, Internet users can be divided into “researchers,” “consumers,” “expressers,” and “entertainers” (9). Different ways of using the Internet will produce different social benefits, and recreational and social use of the Internet have more significant effects on health, affecting life satisfaction and well-being (7, 10, 11). Internet use is affected by individual, cultural, technological and social factors such as gender, race, registered residence, geographical location and socio-economic status (12), which can directly affect health. According to the empirical analysis results of China's typical micro database (Charles), self-rated health status is significantly related to Internet use, and narrowing the digital divide can reduce health inequality (12). Problematic Internet Use (PIU) caused by COVID-19 is even more serious. There are country differences in this situation, which will aggravate loneliness, damage self-esteem, and cause psychological distress. It is necessary to be alert to adverse consequences for health caused by differences in using Internet. (13). The above research conclusions show that more and more scholars are paying attention to the important impact of the Internet on population health, but there are still some theoretical differences in the relationship between the two (14, 15), especially since few studies are focusing on the effect of the IUG on health, and only a few scholars have proposed that narrowing the digital divide can reduce health inequality (16).

The Internet can affect health from multiple domains, one of which is information (17, 18). Through the analysis of Knowledge Gap Theory, it is found that the Digital Divide causes unequal opportunities for information interaction, and the ability to search and utilize information will affect the use of the Internet by affecting human capital, thereby deepening the IUS (18). The Internet brings about the explosive growth of information, which greatly improves the utilization effect of information, especially medical and health information, and makes it develop into one of the important ways to obtain health information and knowledge (19), forming the “Internet information dependence” (IID) of health capital. IID can affect both the digital divide and population health. On the one hand, the development of Internet information technology can enhance the digital dividend, and it may also cause an information gap due to the difference in the possession and utilization of information technology, thereby increasing the digital divide (20). On the other hand, the rapid ingestion of Internet information can improve the ability to participate in information, meet information needs, enhance self-esteem experience and alleviate the fear of the unknown (21). IID has two dividing dimensions, namely, the availability of information and the usefulness of information (22). The former affects health behaviors through as much information as possible, and the latter ultimately affects health decisions through information screening. The improvement of Internet health information literacy is an important condition to ensure mental health. However, the low threshold for Internet information access will cause information risks, and the diversity, extensiveness, misleading and uncertainty of information will cause anxiety and tension to a certain extent, and affect health (20). Based on the above analysis, it is found that IID can affect health through direct and indirect paths, but its indirect effect paths are more diverse, and few people have paid attention to the positive role of IID in the negative effect of IUS on health. The collaborative research on the three is relatively scarce and needs to be further deepened from the theoretical level.

At present, the research on the impact of the Internet on health has mostly focused on the use of smartphones and social media (23). The research methods are mostly qualitative surveys such as statistical regression (24) and focus interviews (25), and the key groups of concerning are special groups such as adolescents (26) and the elderly (24). Thus, quantitative research results for developing countries are relatively scarce. This study used the 2018 China Family Panel Studies Database (CFPS) as the research data source extracting relevant measurement indicators such as population health, IUG, IID, etc. According to the data types and characteristics of the questionnaire, Mplus 8.0 software was used to carry out LCA with distal outcomes and mediation test. The findings of this study have important implications for reducing the negative effects of the IUG on population health in low- and middle-income countries.

## Methods

### Data source

The research data came from China's 2018 CFPS cross-sectional database released by the China Social Science Survey Center (*N* = 11086), which covered 31 provinces/cities/autonomous regions of China and had the characteristics of national, systematic, social and continuous, etc. The data is permanently tracked with a two-year survey cycle. Since the nationwide survey in 2010, a total of 8 rounds of data collection have been carried out, including the preliminary survey. It is an important micro-database for studying Chinese social issues. This study selected the content of family questionnaires and adult questionnaires as the main data sources, and the survey content covered multiple dimensions such as income, education, society, medical care, and health.

### Analytic strategy

The data analysis is divided into three stages. The main goal of the first stage is to identify the Internet use subtypes of different users through five Internet use variables which is used as the IUG variable. Then the second stage is to examine individual characteristics, social characteristics, living habits and health differences between the latent classes. The main goal of the third-stage analysis was to observe the mediating effect of IID on the population health pathway of the IUD.

LCA is a method of parameter estimation based on the principle of probability distribution and the joint probability of individuals on explicit variables (27), which can reorganize explicit variables of specific Internet-use behavior into categorical variables of Internet use behavior patterns. The key explanatory variable of this study is the IUG, which consists of five explicit variables, namely the frequency of Internet study, work, social interaction, entertainment and business activities (divided into high-frequency and low-frequency categories according to the questionnaire information), and they are denoted as A, B, C, D, and E, respectively. The corresponding latent variable model is constructed as:


(1) $$\begin{array}{l} \pi_{fghij}^{ABCDE}=\overset{N}{\underset{n=1}{\sum }}\pi_{n}^{X}\pi_{fn}^{A|X}\pi_{gn}^{B|X}\pi_{hn}^{C|X}\pi_{in}^{D|X}\pi_{jn}^{E|X} \end{array}$$

In formula (1), f, g, h, j, and k are the values of five explicit variables corresponding to specific Internet use behaviors, respectively. $\pi_{\text{fghij}}^{ABCDE}$ represents the joint probability of a latent Internet use category model, and $\pi_{\text{n}}^{X}$ is the latent class probability, that is, the probability that a latent class X belongs to class *n*, *n*= 1, 2...N. $\pi_{\text{fn}}^{A|X}$ is the conditional probability, that is, the probability that an individual belonging to the nth latent category responds to the fth level of the observed variable A. The subsequent interpretation of the conditional probability is similar to this, so it will not be explained one by one.

The Bolck, Croon, and Hagenaars (BCH) approach of LCA was used to predict distal outcomes, which modeled all covariates and distal outcomes simultaneously in the final LCA solution (28). This analytical method has significant advantages, which indicates that modal posterior probability assignment is used to reflect respondents to their most likely latent class and performs the subsequent weighted multi-categorical analysis. This stepwise approach can not only provide overall significance testing of associations between latent class membership and outcomes but also perform pairwise differences testing between classes in the means of continuous outcomes (29). In addition, this method can fully consider the influence of covariates on dependent variables so that the effects of latent class membership are controlled by those covariates (30).

Moreover, we used the model constraint function in Mplus to build a mediation model. After controlling for covariates such as individual characteristics, with the latent class variable of IUG as independent variable, we observed IID as intermediary variable, and population health as the dependent variable. We examined whether the mediation effect of Internet information dependence is established.

All models were done by using Mplus version 8 and State 16.

### Variable selection

#### Dependent variable (DV)

The dependent variable is population health. According to the question “how do you think your health status” in the personal questionnaire QP201, the score is 1–5, and the higher the value is, the higher the health level is.

#### Independent variable (IV)

The independent variables mainly analyze two factors, the IUG and IID.

1) Internet use gap (IUG). According to the frequency of Internet study, work, social interaction, entertainment, and business activities, a categorical variable of Internet use with significant explanatory power was constructed, and it was used as an integrated variable for the IUG. Use Mplus 8.0 software to fit the data many times, and compare the fitting effects of different models, then finally determine the best fitting model according to the Log-Likelihood G2, AIC and BIC. Use Entropy (value 0–1) to evaluate the accuracy of the classification, and focus on the values of AIC, BIC, and aBIC. The smaller the values are, the better the fitting effect of the classification results is (31).
2) Internet information dependence (IID). The measurement of IID in previous research was mostly carried out from the perspectives of Internet access such as Internet coverage and mobile phone penetration. A small number of scholars used the time of using Internet to measure the degree of Internet use (32), and some scholars comprehensively analyzed the availability and effectiveness of Internet information (22). This study mainly focuses on the gap and information dependence based on Internet use and focuses more on the usefulness of Internet information. Therefore, the question of QU802 “Importance of the Internet for you to obtain information” is selected as the explanatory variable of IID, and the values are assigned from 1 to 5, and the degree of importance increases gradually.

#### Control variable (CV)

To minimize the problems of endogeneity and heteroscedasticity, the study further selected factors that may affect health from different dimensions such as individual characteristics, social characteristics and living habits, as covariates in the analysis framework. Among them, individual characteristics include variables such as gender, age, marital status, years of education and chronic disease prevalence, and social characteristics include variables such as income, employment status and family size. Drinking, exercise and smoking are used as indicators of living habits.

The basic description and statistics of the variables are shown in Table 1. In order to avoid the possible multicollinearity of the multiple explanatory variables of the cross-sectional data from affecting the regression results as much as possible, the Tolerance (Tol) and the Variance Inflation Factor (VIF) were tested respectively, and it was found that the Tol values were much >0.1 and VIF values were all <5, which indicated that the selected explanatory variables have passed the multicollinearity test and can be further analyzed.

**Table 1:** Variable description and descriptive statistics.

**Variable**	**Name**	**Description**	**Mean**	**SD**
Health	Health	Score ranges: 1–5. The larger score, the healthier	3.015	1.183
IUGV	First class	Light-life users, *N* = 1061, C1	0.10	0.294
	Second class	All-around users, *N* = 1980, C2	0.18	0.383
	Third class	Functional users, *N* = 1239, C3	0.11	0.315
	Fourth class	Pure-life users, *N* = 6806, C4	0.61	0.487
IIDV	INT infomation	Importance of the Internet for you to obtain information Value ranges: 1–5	3.185	1.6154
ICV	Gender	Gender: Male = 1, Female = 0	0.50	0.500
	Age	Age: actual age. Value ranges: 16–96	45.12	14.869
	Marriage	marital status: with spouse = 1, without spouse = 0	0.81	0.396
	Education	Years of education, from illiterate to doctorate. Value ranges: 0–22	8.33	4.732
	Chronic	chronic disease: Do you have the chronic disease? Yes = 1, No = 0	0.14	0.345
SCV	Employ	Working status: employed = 1, unemployed = 0	0.82	0.383
	Family number	Family size: number of family members. Value ranges: 1–17	4.09	1.997
	Income	Income: logarithm of per capita household income (annual income/family size)	9.490	0.998
LHV	Exercise	Exercise: the frequency of physical exercise in the last week. Value ranges: 0–50	2.55	3.271
	Smoke	Smoking: Smoking in the past month? Yes = 1, No = 0	0.68	0.465
	Drink	Drinking: 3 times per week in the past month? Yes = 1, No = 0	0.84	0.368

IUGV, Internet use gap variable; IIDV, Internet information dependence variable; ICV, Individual characteristic variables; SCV, Social characteristic variables; LHV, Living habits variables; same below.

According to the results of Table 1, the selected survey sample's health level is high as a whole (*M* = 3.015, SD = 1.183), and there is only a small part of the chronic disease (*M* = 0.14, SD = 0.345), of which Pure-Life uses group is the most (*N* = 6806, Category Probability = 6806/11086 = 61.39%) and Light-life users' group is the least (*N* = 1061, Category Probability = 1061/11086 = 61.39% = 9.57%). At the same time, the sex ratio of the investigated population is balanced (*M* = 0.50, SD = 0.500), of which the age is relatively high (*M* = 45.12, SD = 14.869), and most of them are married (*M* = 0.81, SD = 0.396) and working (*M* = 0.82, SD = 0.383). However, their education level is low, and the average years of schooling is only 8.33, most of them lack healthy habits, and the average weekly exercise frequency is only 2.55. The proportion of people who smoke (*M* = 0.68, SD = 0.465) and drink (*M* = 0.84, SD = 0.368) is relatively large.

## Results

### Latent class identifification

**Figure 1:**
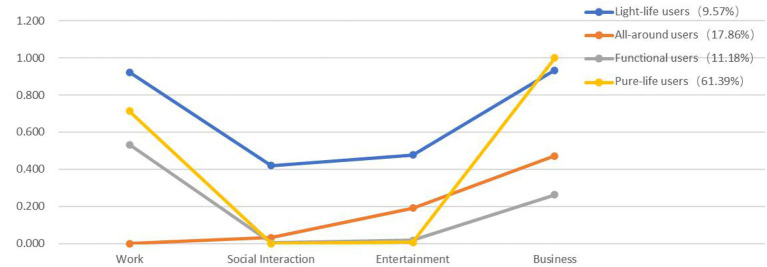
Item response probabilities and prevalence for the four-class model.

The potential category analysis of the IUG is mainly completed through the Mplus 8.0 software. See Table 2 for the specific analysis results. It can be seen from the analysis results that the likelihood ratio statistic G^2^ gradually decreases with the increase of the number of categories, and the degree of freedom level increases, but the type4 has the smallest AIC (44302.367), BIC (44470.579) and aBIC (44397.487) values, and the Entropy value (0.735) is higher than that of the type 5 (0.718), so the study considers the latent category model of type 4 to be the optimal category model. Based on this, the study further analyzes the latent category probability and conditional answer probability of different Internet use classifications, and names the corresponding Internet use types according to the corresponding conditional answer probability. The results are shown in Table 3 and Figure 1. Users in category 1 are weak in other functions except for social and entertainment, so they are named “light-life users.” Similarly, users in category 2 with a high frequency of five functions are named “all-around users.” Users in category 3 have relatively high frequencies except for the low frequency of working, which is named “functional users.” Users in category 4 have a very low frequency of functional usage except for the very high frequency of social entertainment, and they are named “pure-life users.” The latent category probabilities of the four types of users are 9.57, 17.86, 11.18, and 61.39%, respectively. Different types of Internet users not only reflect differences in individual Internet use but also directly reflect the Internet digital divide. It is proved that differences in Internet use patterns create an IUG.

**Table 2:** Latent category model indicator evaluation of internet use categories.

**Type**	**Likelihood ratio statistic *G^2^***	***P*** **Value**	***AIC***	***BIC***	***aBIC***	**Entropy**	**Degrees of freedom**
Type 1	−25116.997	0.000	50243.993	50280.561	50264.672	0	5
Type 2	−22669.355	0.000	45360.711	45441.16	45406.203	0.841	11
Type 3	−22196.292	0.000	44426.584	44550.914	44496.893	0.775	17
Type 4	−22128.184	0.000	44302.367	44470.579	44397.487	0.735	23
Type 5	−22108.162	0.000	44374.332	44486.422	44398.263	0.718	29

**Table 3:** Latent category probability and conditional answer probability of internet use model (%).

**Latent category**				**Light-life users**	**All-around users**	**Functional users**	**Pure-life users**
Category probability				9.57%	17.86%	11.18%	61.39%
Conditional probability	Study	Internet use frequency	Low↓	0.929	0.067	0.408	1.000
			High↑	0.071	0.933	0.592	0.000
	Work		Low↓	0.923	0.000	0.532	0.713
			High↑	0.077	1.000	0.468	0.287
	Social Interaction		Low↓	0.419	0.031	0.002	0.000
			High↑	0.581	0.969	0.998	1.000
	Entertainment		Low↓	0.478	0.190	0.017	0.007
			High↑	0.522	0.810	0.983	0.993
	Business		Low↓	0.934	0.471	0.262	1.000
			High↑	0.066	0.529	0.738	0.000

### Class characteristics

Individual characteristics, social characteristics and living habits for the four classes are displayed in Table 4. We tested whether classes differed in individual characteristics, social characteristics, living habits, IIDV and health. Smoking status and IID did not differ significantly among potential subcategories of Internet use. Different Internet use categories have significant between-group differences in multiple variables and have their characteristics.

**Table 4:** ICV, SCV, LHV, health and IIDV of latent classes.

**Variable**		**M(SD)/Prob(OR)**			
		**Light-life users (C1)**	**All-around users (C2)**	**Functional users (C3)**	**Pure life users (C4)**
	**Number**	**1061**	**1980**	**1239**	**6806**
ICV	^B^Gender^a^**= 1	0.531 (1.225)	0.503 (1.096)	0.516 (1.154)	0.480 (1.000)
	^A^Age^a^^***^	44.765 (0.421)	40.607 (0.480)	41.125 (0.426)	47.511 (0.204)
	^A^Edu^a^^***^	7.890 (0.146)	10.073 (0.171)	8.461 (0.146)	7.958 (0.062)
	^B^Marriage^a^^***^= 1	0.883 (1.747)	0.743 (0.671)	0.777 (0.810)	0.811 (1.000)
	^B^Chronic^a^ ^***^= 1	0.136 (0.884)	0.118 (0.757)	0.115 (0.735)	0.151 (1.000)
SCV	^A^Family number^a^^***^	4.443 (0.065)	4.367 (0.068)	4.645 (0.066)	3.780 (0.026)
	^B^Employ^a^^***^= 1	0.921 (0.009)	0.855 (1.812)	0.903 (2.858)	0.766 (1.000)
	^A^Income^a^^***^	9.349 (0.032)	9.599 (0.036)	9.515 (0.032)	9.489 (0.013)
LHV	^A^Exercise^a^^***^	2.220 (0.102)	2.633 (0.103)	2.322 (0.102)	2.667 (0.045)
	^B^Smoke = 1	0.670 (0.959)	0.715 (1.186)	0.689 (1.047)	0.679 (1.000)
	^B^Drink^a^***^^= 1	0.843 (0.992)	0.875 (1.286)	0.780 (0.653)	0.844 (1.000)
IIDV	^A^IIDV^a^^***^	3.070 (0.050)	3.571 (0.051)	3.555 (0.048)	3.002 (0.022)
Health	^A^ *Health* ^***^	3.089 (0.040)	3.151 (0.037)	3.070 (0.035)	2.948 (0.016)

Note: Significant differences between all groups, where

*** *p* < 0.001.

A Indicates that the variable is continuous and reports M (SE).

B Indicates that the variable is categorical and reports Prob (OR).

a Indicates significant differences between C1 and the rest of the groups. *M*, means; OR, Odds Ratio.

Based on research needs, focus on observing whether there are significant differences in the health level and IID of different potential categories of Internet users. According to LCA with distal outcome, there are significant differences in the health level (C1: M = 3.089, SE = 0.040; C2: M = 2.948, SE = 0.016; C3: M = 3.070, SE = 0.035; C4: M = 2.948, SE = 0.016) and IID (C1: M = 3.070, SE = 0.050; C2: M = 3.571, SE = 0.051; C3: M = 3.555, SE = 0.048; C4: M = 3.002, SE = 0.022) of different potential categories of Internet use. Furthermore, the characteristics of C2 and C4 users are the most prominent. C2 users' educational level educational level (M = 10.073, SE = 0.171) and income level (M = 9.599, SE = 0.036) are higher than other categories of Internet users, but the age is lower (M = 40.607, SE = 0.480). C4 users have the longest age (M = 47.511, SE = 0.204), but their family size (M = 3.780, SE = 0.026) was minimum.

### Intermediary effect validation

We further examined whether differences between the functional user's group and the three internet use groups in IID explained differences in health. Thus, multi-categorical weighted mediation analyses (33) were performed to determine whether differences in the degree of dependence on internet information between each internet use class and the functional users class accounted for health of the IUG after controlling for covariates.

**Figure 2:**
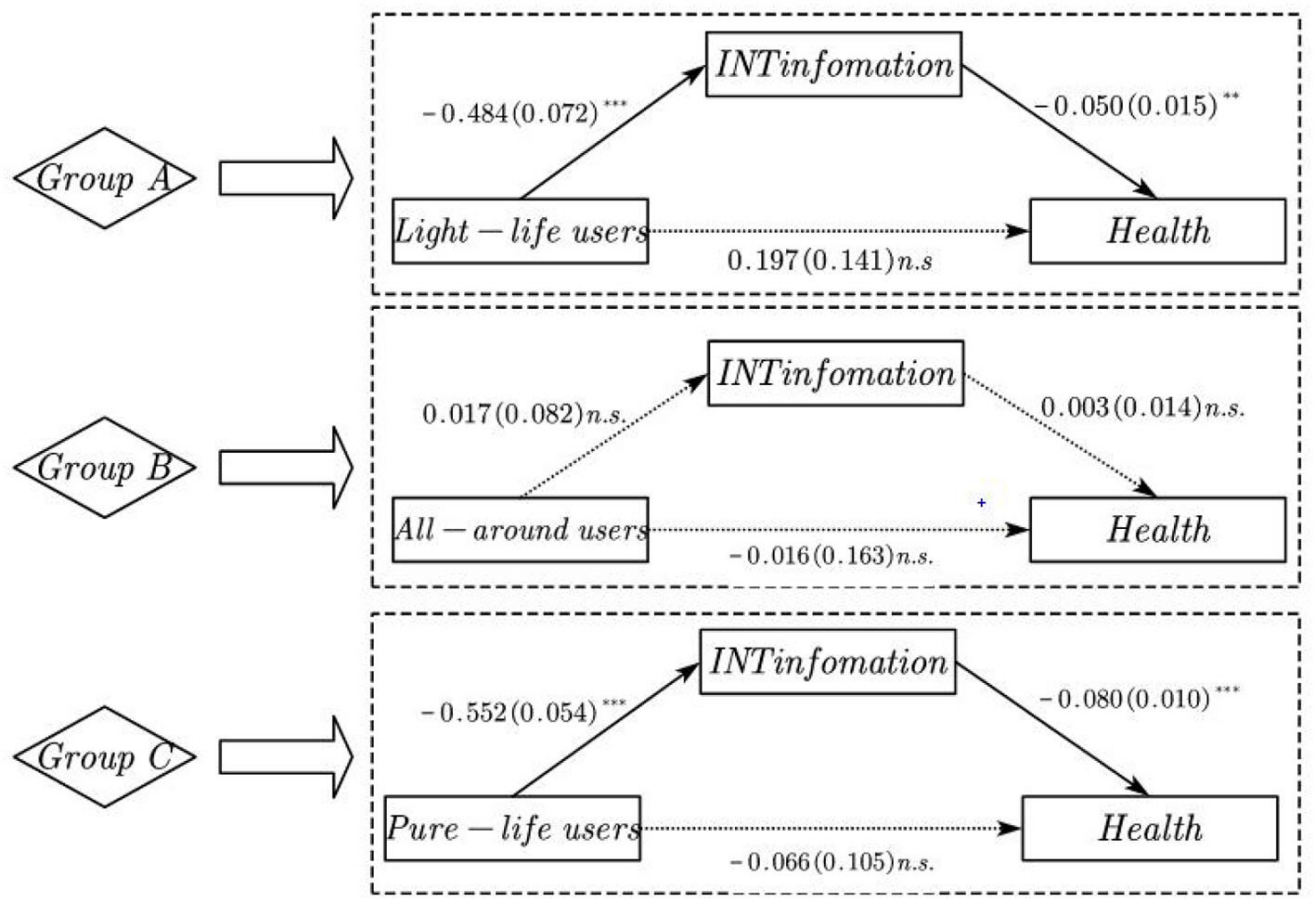
Multigroup mediation model relative to the Functional users (C3). Note: **(Group A)** Comparison of Light-life users class (C1) against the Functional users class (C3). **(Group B)** Comparison of All-around users class (C2) against Functional users class (C3). **(Group C)** Comparison of Pure-life users class (C4) against Functional users class (C3). Significant directional paths are represented in solid lines and non-significant paths in dashed lines. Our statistical model included health at covariates (*P < 0.05. **P < 0.01. ***P < 0.001).

Tests of indirect effects via IID were examined in the same model (see Figure 2 and Table 5). As seen in Figure Group A, for internet users of Light-life (C1), higher dependence on internet information (*b*_*indirecteffect*_ = −0.050, SE = 0.18, Est./SE = −3.264, *P* = 0.001), partially explained differences in health relative to the Functional users class. However, the effect of class on health was not significant after adding indirect paths (*b*_*indirecteffect*_= 0.197, SE = 0.141, Est./SE = 1.399, *P* = 0.162). Although health was not significantly higher for C2 than for C3 after accounting for all other measures, the extent to which health was higher in All-around users' class was not accounted for by different degree of dependence on internet information. As seen in Figure Group B, for internet users of All-around (C2), evidencing greater health was not explained by the dependence on internet information (*b*_*indirecteffect*_ = 0.003, SE = 0.014, Est./SE = 0.202, *P* = 0.840) in the Functional users group. Consistent with Group A, the effect of class on health was not significant after adding indirect paths (*b*_*indirecteffect*_ = −0.016, SE = 0.163, Est./SE = −0.099, *P* = 0.921). As seen in Figure Group C, in Pure-life users group (C4), lower dependence on internet information relative to the Functional users group fully explained the extent to which people had reduced health (*b*_*indirecteffect*_ = −0.080, SE = 0.010, Est./SE = −8.412, *P* = 0.000). However, the effect of class on differences in health was not significant after adding indirect paths (*b*_*indirecteffect*_ = −0.066, SE = 0.105, Est./SE = −0.631, *P* = 0.528).

**Table 5:** Class differences relative to the functional users (C3).

**Variable**	**M(SE)**		
	**Light-life users(C1)**	**All-around users(C2)**	**Pure life users(C4)**
Take C3 as the reference group			
Class(x) - Health	0.197 (0.141)	−0.016 (0.163)	−0.066 (0.105)
Class(x) - IIDV	−0.484 (0.072)^***^	0.017 (0.082)	−0.552 (0.054)^***^
IIDV - Health	−0.050 (0.015)^**^	0.003 (0.014)	−0.080 (0.010)^***^

ICV, SCV, LHV are controlled, and results for one dependent variable control for theother dependent variables (i.e., class differences in health for IIDV).

ICV, Individual characteristic variables; SCV, Social characteristic variables; LHV, Living habits variables; IIDV, Internet information dependence variable.

*P < 0.05,

** *P* < 0.01,

*** *P* < 0.001.

## Discussion

The Internet is the core technical element and production element for the life and development of modern people and affects health. According to the empirical analysis of the 2018 CFPS database, the research analyzes the mechanism of the IUG and IID on health. First of all, by analyzing the latent categories of various ways of using the Internet, the users who use the Internet can be divided into light-life users, all-around users, functional users, and pure-life users. There are obvious differences in the latent category probabilities of different user types, which reflect the IUG, indicating that IUG exists objectively. Different latent categories of Internet users present certain individual and social characteristics. Secondly, the IUG has a direct effect on health. According to the results of the BCH approach for LCA with distal outcomes, it is found that there are significant differences in the health level of Internet users in different potential categories(C1: *M* = 3.089, SE = 0.040; C2: *M* = 2.948, SE = 0.016; C3: *M* = 3.070, SE = 0.035; C4: *M* = 2.948, SE = 0.016). This shows that theIUG formed by different Internet usage does have different effects on population health. In addition, by analyzing the mediating effect of the model constraint function in Mplus, it is found that the mediating effect of Internet information dependence on the healthy path of the Internet use gap exists.

Based on the above research conclusions, corresponding policy recommendations are put forward. First, pay attention to the diversified, life-oriented, and balanced development of Internet use. When analyzing the classification characteristics of potential subclasses formed by different Internet use situations, it is found that C2 Internet users have higher social, economic status and health status, while C4 Internet users have the opposite, which indicates that C4 Internet users have certain limitations and limit the positive impact of the Internet on population health. It is necessary to consider the production and life functions of Internet use, maximize the social benefits of the Internet's digital dividends, be alert to the negative effects of the IUG on health, and prevent the Internet's “technological addiction,” “entertainment addiction” and “social addiction.” Second, make good use of the positive guiding function, transmission function, and interactive function of Internet information channels. The intermediary effect test proves that the intermediary effect of IID on the healthy path of the impact of the IUG does exist, which indicates that the information transmission function of the Internet is very important. The Internet is an epoch-making change in information dissemination. It promotes the global dissemination and sharing of information and affects people's personality psychology, value orientation, and way of life in an all-around way. Opportunities and challenges of Internet information dissemination should be treated with caution. On the one hand, we should build a strong supervision network for Internet information, strictly punish the dissemination of harmful information and Internet information crimes, and guide correct, positive, and healthy network information to occupy the network position. On the other hand, pay attention to the guidance of ideology and the propaganda on the correct use of Internet information, and improve the people's information literacy and information network screening ability. Third, pay attention to and protect marginalized groups in the digital age. C2 Internet users are the dominant group of Internet users. They are generally younger and better at using the Internet to create health benefits and social benefits, However, C4 Internet users' high age and low education levels restrict their ability to use the Internet, resulting in low health benefits and social benefits. Due to insufficient Internet accessibility and technical requirements for usability, disadvantaged groups in digital development such as the elderly, female groups, and rural groups are formed, making it difficult to absorb digital dividends and even causing double cumulative disadvantages. For such disadvantaged groups, individualized and tendentious Internet resource platform development and technical support are needed to ensure the fairness of opportunities and the fairness in their Internet use.

## Advantage

Research has certain innovations and advantages. First, the research object is typical, and the research data is scientific. Select China, which has the characteristics of the largest developing country and the largest number of Internet users, as the research object, and select the 2018 CFPS database as the data source (*N* = 11086). The database is a typical representative micro-database in China, with a wide range of survey objects. The survey content includes social fields, people's livelihood fields, and individual characteristics. It provides a first-hand data source for empirical analysis and ensures the integrity and feasibility of the research program. Second, the research perspective is innovative. The Internet is one of the social determinants that affect health (3), and there are still some theoretical differences in the relationship between the Internet and health. Previous studies have focused on the effects of social media usage and information technology development on health. Few studies have paid attention to the different effects that the diversity of Internet use may have on health. The research studies the effect of the Internet on health based on the IUG and provides new research thoughts and research strategies for related topics. Third, The BCH method was used to perform latent class modeling with a continuous distal outcome, which is robust to violations of analysis model assumptions in comparison to other stepwise approaches (34). Furthermore, this approach also allows the inclusion of covariates so that the effects of latent class membership are controlled by those covariates (35). In addition, building a mediation model about Internet information dependence through the model constraint function in Mplus is more perfect than the simple potential category analysis, which makes the research contains more completely.

## Limitation

This research also has a few limitations. Firstly, the research data in this study are cross-sectional data. There may be a time lag in the effect of the Internet on health, which affects the explanatory power of the regression results. At the same time, the selection of research and analysis indicators is based on the principle of convenient sampling, which is subjective, and there are potential psychological characteristics to confuse the relationship between independent variables and dependent variables (36). In addition, the research data has a certain time lag. Limited by the availability of data, the research can only use the latest research data (2018CFPS) currently available for research, which has a certain time difference from the current time, and may be different from the current actual situation, which needs to be released at a later stage. Timely update data when the latest data is available to further improve research recommendations.

## Conclusions

The COVID-19 epidemic continues to spread around the world, further creating an urgent health need (37). However, the spread of the COVID-19 epidemic is accompanied by a surge in the frequency of Internet use. The resulting problems of Internet addiction and overuse may pose a major public health threat. It is necessary to be alert to the adverse consequences caused by differences in Internet use (13). The research results show that there are significant differences in the health level of different potential types of Internet users. At the same time, the individual characteristics, social characteristics and living habits of different groups also have obvious group differences. In addition, Internet information dependence does play a mediating role in the process of Internet use gap affecting population health. It inspires for developing countries to improve the level of national health. Future research should further examine the time lag of the impact of the IUG and IID on health.

## Data availability statement

The raw data supporting the conclusions of this article will be made available by the authors, without undue reservation.

## Author contributions

Study design, data cleansing, and statistical analysis: YH and LZ. Supervision: LZ and XX. Writing—original draft: LZ and JuL. Writing—review editing: YH and JiL. Financial support: LZ. All authors contributed to the article and approved the submitted version.

## Funding

This study was supported in parts by the National Natural Science Foundation of China General Program (71974064) Value-oriented chronic disease outpatient insurance payment model construction and support strategy research; 2020 province the Research and Practice Innovation Plan for Graduate Students (KYCX20_3053) Research on the Response Strategies of Medical Security Participating in the Management of Public Health Emergencies.

## Conflict of interest

The authors declare that the research was conducted in the absence of any commercial or financial relationships that could be construed as a potential conflict of interest.

## Publisher's note

All claims expressed in this article are solely those of the authors and do not necessarily represent those of their affiliated organizations, or those of the publisher, the editors and the reviewers. Any product that may be evaluated in this article, or claim that may be made by its manufacturer, is not guaranteed or endorsed by the publisher.

## Abbreviations

CFPS: China family panel studies database
IUG: internet use gap
IID: internet information dependence
CV: control variable
DV: dependent variable
IV: independent variable
ICV: individual characteristic variables
DE: direct effect
IE: intermediary effect
LCA: latent class analysis
PIU: problematic internet use
MP: mediation percentage
LHV: living habits variables
SCV: social characteristic variables.
